# Landscape heterogeneity rather than crop diversity mediates bird diversity in agricultural landscapes

**DOI:** 10.1371/journal.pone.0200438

**Published:** 2018-08-01

**Authors:** Sarah Redlich, Emily A. Martin, Beate Wende, Ingolf Steffan-Dewenter

**Affiliations:** Department of Animal Ecology and Tropical Biology, Biocenter, University of Würzburg, Würzburg, Germany; Leiden University, NETHERLANDS

## Abstract

Crop diversification has been proposed as farm management tool that could mitigate the externalities of conventional farming while reducing productivity-biodiversity trade-offs. Yet evidence for the acclaimed biodiversity benefits of landscape-level crop diversity is ambiguous. Effects may strongly depend on spatial scale and the level of landscape heterogeneity (e.g. overall habitat diversity). At the same time, contrasting within-taxon responses obscure benefits to specific functional groups (i.e. species with shared characteristics or requirements) if studied at the community level. The objectives of this study were to 1) disentangle the relative effects of crop diversity and landscape heterogeneity on avian species richness across five spatial scales ranging from 250 to 3000 m radii around focal winter wheat fields; and 2) assess whether functional groups (feeding guild, conservation status, habitat preference, nesting behaviour) determine the strength and direction of responses to crop diversity and landscape heterogeneity. In central Germany, 14 landscapes were selected along independent gradients of crop diversity (annual arable crops) and landscape heterogeneity. Bird species richness in each landscape was estimated using four point counts throughout the breeding season. We found no effects of landscape-level crop diversity on bird richness and functional groups. Instead, landscape heterogeneity was strongly associated with increased total bird richness across all spatial scales. In particular, insect-feeding and non-farmland birds were favoured in heterogeneous landscapes, as were species not classified as endangered or vulnerable on the regional Red List. Crop-nesting farmland birds, however, were less species-rich in these landscapes. Accordingly, crop diversification may be less suitable for conserving avian diversity and associated ecosystem services (e.g. biological pest control), although confounding interactions with management intensity need yet to be confirmed. In contrast, enhancement of landscape heterogeneity by increasing perennial habitat diversity, reducing field sizes and the amount of cropland has the potential to benefit overall bird richness. Specialist farmland birds, however, may require more targeted management approaches.

## Introduction

Agrochemical inputs, intensive crop rotations and removal of non-crop habitats directly and indirectly affect resource availability and habitat diversity in agroecosystems. As a result, biodiversity and ecosystem services decline [1,2]. Agricultural extensification (the use of less intensive farming methods) could mitigate these trends. To date, biodiversity conservation efforts primarily focus on extensification measures that facilitate the often-pronounced relationship between taxonomic biodiversity and the amount and diversity of non-crop habitats. However, apparent biodiversity-productivity trade-offs lower the profitability and uptake of extensification approaches such as flower strip plantings or set-asides, which often require arable land to be taken out of production [3].

Crop diversification (i.e. increasing the number and evenness of crops grown within a given landscape) has been proposed as an alternative extensification strategy that could reduce the negative effects of conventional farming without jeopardizing productivity goals [4]. Like non-crop habitat diversity, landscape-level crop diversity can play a vital role in sustaining biodiversity and ecosystem services. By providing a variety of complementary resources and habitats in space and time, more species with multiple and seasonal extended resource requirements or different niches can persist (complementation or niche differentiation effects) [4–9]. These additional resources are particularly relevant in intensively farmed landscapes, where non-crop elements such as seminatural habitats are often deteriorated beyond functional importance [10].

Yet evidence for the benefits of landscape-level crop diversity (hereafter ‘crop diversity’) is ambiguous, especially with respect to birds. Birds, in particular farmland birds, contribute a range of essential ecosystem services such as pest control (herbivore and weed seed removal, [2,11]) and nutrient cycling [12]. The composition of bird assemblages relates to the quality, structural diversity, disturbance level and food availability of cropping systems at local and landscape scales, thereby giving insights into the state of plant, insect and vertebrate diversity as a whole [13]. Yet previous findings showcase a range of very context-specific and opposing effects [14–18].

Crop diversity benefits may vary depending on the spatial scale considered [19–22] and can be confounded by or interact with landscape heterogeneity [7,18,23]. Here, we define landscape heterogeneity as an array of strongly interrelated components of configuration (mean patch size) or composition (perennial habitat diversity, seminatural habitat cover) that do not relate to the type of crops grown within the landscape. Choosing an inadequate spatial scale or missing correlations with landscape heterogeneity aspects could therefore result in false positive, negative or absent effects of crop diversity. At the same time, crop diversity effects may not equally apply to all bird species, owing to different resource, habitat and nesting preferences of specific functional groups (i.e. species with shared characteristics or requirements), so that individual responses could be masked in whole community analysis [17,24–26]. Whether effects are found may also depend on the choice of crop diversity index (i.e. which crops are included or whether they are grouped) [18]. As most studies have been restricted to crop diversity estimates based on a limited number of crops [22,27–29], single-species responses [30,31], subsets of the whole community (e.g. farmland birds, [17,18]), or one spatial scale [14,29], this could explain some of the contrasting crop-bird diversity patterns observed.

In this study, we explore the relationship between bird richness and crop diversity, while uncovering factors mediating or limiting benefits for bird communities in agroecosystems. To disentangle crop diversity effects from landscape heterogeneity, 14 sites were selected along two independent gradients of crop diversity and perennial habitat diversity (here used as proxy for landscape heterogeneity). At each site, landscape variables were calculated for five spatial scales (250m, 500m, 1000m, 2000m, 3000m). Opposed to previous studies, we use a crop functional diversity index based on all arable crops grown within the different landscapes. Using bird surveys, we distinguished between influences on the whole bird community, and four functional groups (defined by ‘feeding guild’, ‘habitat preference’, ‘nesting behaviour’ and ‘conservation status’), while posing four hypotheses: First, we expected a positive association between crop diversification and overall bird species richness (complementation or niche differentiation effects, [4]). Second, we anticipated varying responses of different functional groups such as endangered vs. non-threatened species [24]. Third, we tested the hypothesis that crop diversity effects on the whole community and functional groups depend on the level of landscape heterogeneity (intermediate landscape complexity hypothesis [23]) or, fourth, the spatial scale considered [21].

The landscape-level diversity of annual arable crops is associated with high spatial and temporal variability. Crop diversity therefore represents a flexible and adaptable component of a farm, which increases its utility as targeted biodiversity enhancement measure [32]. Here, we shed new light on the possibilities and context-dependencies of crop diversity as conservation tool by considering functional group identity, landscape context and spatial scale.

## Material and methods

### Study region and field selection

Fieldwork was carried out in 2014 in a c. 25 km by 40 km area near Würzburg /Germany (49°47`N, 9°57`E). The intensively cultivated region is dominated by cereals, sugar beets, maize and oil crops, and home to a number of red-listed bird species [33]. Here, 14 focal winter wheat fields were selected along gradients of crop diversity at various scales. Focal fields were at least 1000 m apart (range 1012 m to 2560 m) and selected to have structurally similar field margins (simple grass margins).

### Crop diversity

Resource complementation effects rely on the presence of functionally different plant types [4,18]. Indices estimating diversity based on a large number of crops with similar structure, resources and ecological functions (e.g. wheat, barley, triticale) may therefore overestimate the functional diversity. However, the assignment of specific functions to crops strongly depends on preferences of individual study organisms, which makes this approach particularly difficult in whole community studies. In addition, the inclusion of only a subset of main crops such as cereals, maize and rotational grasslands—as done in previous studies [22,27–29]–may mask important crop diversity effects of less prominent functional crop groups. Based on these considerations, we therefore used all arable crops grown within the study region to create 12 crop categories (Table 1) according to the structural similarity and relatedness of the crops [18,34]. Landscape-level crop diversity (“CropDiv”) was then calculated as Shannon Wiener index in the ‘vegan’ package in R [35] for five spatial scales (250, 500, 1000, 2000 and 3000 m radius around a centroid placed halfway between the two bird observation points, S1 Table). Scales were chosen based on known home ranges of birds, and previous research. The regional agricultural land-use data for 2014 was obtained from the Bavarian State Ministry of Nutrition, Agriculture and Forestry. To assess the risk of underestimating crop diversity using this grouping approach, all analyses were repeated using crop species diversity based on 58 arable crops. The results did not change, but model fit was lower. This supports the use of crop functional rather than crop species diversity [18].

**Table 1:** Description of landscape parameters and species richness variables.

		Min	1st Q	Median	Mean	3rd Q	Max	Description
*Landscape parameters*								
	CropDiv^a^	0	0.84	1.05	1.01	1.21	1.48	Shannon index calculated from the proportional cover of twelve crop types: cereals (excluding grain maize), 1- or 2-year old fallows, flowers and ornamental plants, temporary grassland and green fodder (green maize), legumes, maize, oilseed and fibre crops (excluding rape), rape and turnips, root crops, sunflowers, vegetables, other industrial crops
	LandHet^a^	0.05	0.44	0.71	0.68	0.9	1.32	Due to the high correlation of variables representing aspects of landscape heterogeneity (see text), perennial habitat diversity was used as proxy for the level of heterogeneity in the surrounding landscapes. LandHet was calculated as Shannon index using the proportional cover of six perennial non-crop habitat types: forest, seminatural habitat (orchard meadows, hedgerows, forest edges, field margins, old fallows), settlement, water, perennial crops, extensive permanent grassland. Landscapes with high LandHet also had smaller field sizes, less cropland and more seminatural habitat
*Species richness*^b^								
	Total (63)	15	20	22.5	22.4	25.7	31	Total number of bird species in landscapes. Data obtained from point counts, excluding flocks of birds passing fields.
	**Feeding guild**							
	Insectivore (35)	8.0	14.2	15.5	15.6	17	23	Insect content of diet >60% (including macroinvertebrates)
	Granivore (12)	1	1.3	2.5	2.3	3	4	Seed and plant content of diet >60%
	Carnivore (7)	1	2	2	2.2	2	5	Vertebrate content of diet >60%
	Omnivore (9)	1	1	2	2.4	3	4	Mixed plant and invertebrate diet
	**Conservation status**							
	Least concern (42)	10	15.2	17	16.8	19	21	Bird species with stable population sizes
	Vulnerable (10)	1	2	2	2.9	3	6	Bird species listed as vulnerable in the Bavarian Red List
	Endangered (11)	1	2	3	2.9	3.8	5	Bird species listed as endangered, critically endangered, regionally extinct, very rare or geographically restricted
	**Habitat preference**							
	Farmland (25)	5	7	9	9.2	11	14	Nesting and/or foraging predominantly in cropland
	Non-farmland (38)	6	12	13	13.1	15.8	19	Nesting and/or foraging predominantly in non-crop habitat
	**Nesting behaviour**							
	Crop nester (8)	1	2	2	2.5	3	5	Subset of farmland birds nesting in cropland
	Non-crop nester (17)	3	4.5	6	6.6	7.8	12	Subset of farmland birds nesting in non-crop habitat

Summary statistics of landscape parameters and species richness variables. For landscape parameters crop diversity (“CropDiv”) and perennial habitat diversity (LandHet, the proxy for landscape heterogeneity) summary statistics are averaged across all study sites (n = 14) and spatial scales (n = 5). For total and functional group richness, values are averaged across study sites.

^a^ For summary statistics of individual spatial scales (250, 500, 1000, 2000 and 3000m) see S1 Table

^**b**^ Total number of bird species across all study sites for the whole bird community and individual functional groups shown in brackets

### Landscape heterogeneity

In contrast to CropDiv, other influential landscape aspects such as the diversity of non-crop perennial habitats, arable field size, the proportion of cropland and seminatural habitat cover can be viewed as indicators of landscape heterogeneity not directly related to the type of crop grown. These aspects of landscape composition and configuration can potentially confound crop diversity effects [4]. During field selection, correlations with CropDiv were therefore kept to a minimum (S1 Table). However, as these variables were highly correlated amongst themselves (S1 Table), only perennial habitat diversity (hereafter “LandHet”, correlation with CropDiv *r* = 0.05–0.4, S1 Table) was used in our analysis as proxy for the overall level of landscape heterogeneity. Accordingly, heterogeneous landscapes had a high perennial habitat diversity, a high proportion of seminatural habitat, low cropland cover and small arable field sizes. The indicator variable LandHet was calculated as Shannon Wiener index of six perennial habitat types (Table 1), which were digitized in ArcMap v. 10 [36] using official digital topological maps ATKIS DTK 25 (Bayerische Vermessungsverwaltung).

### Bird observations

Birds were surveyed four times between May and July 2014 next to the focal winter wheat fields. The observation period was chosen to coincide with the major breeding season of birds in Germany. Each survey comprised two 10-minute point counts, one located in the open grass field margin, the other close to the nearest non-crop habitat, the type of which was also recorded (shrubs, forest, other). Distance between field margins and nearest non-crop habitat ranged between 20 to 100 m, the midpoint acted as centroid for landscape calculations. Fields were visited from 4:30 am to 9 am in the morning, or 5 pm to 8:30 pm in the evening. The order and time of visits was randomized. All birds seen or heard within a radius of 100 m were recorded [37]. No distinctions were made between birds breeding or foraging. Surveys were not conducted during windy or rainy weather. All observations were done by a single observer (B.W.), and care was taken not to double-count individual birds.

Bird richness was then based on all species recorded in each landscape during the four visits, with field and non-crop point counts pooled per site. Groups of flocking birds crossing the fields were not included in species richness calculations. Observed and rarefied species richness (estimated in the ‘vegan’ package in R) were highly correlated (Pearson’s *r* = 0.93), suggesting that sampling effort was sufficient. Bird species richness was further partitioned into functional groups (Tables 1 and S2) based on overall ‘habitat preference’ and ‘feeding guild’ [24,38,39]. Birds primarily foraging in cropland may also vary in their sensitivity to crop and non-crop components of agroecosystems owing to their ‘nesting behaviour’ [17,18]. We consequently used the farmland bird subset to distinguish between crop and non-crop nesting species. Finally, we assessed the responsiveness of endangered and vulnerable species in comparison to those with least conservation concern (‘conservation status’ as indicated by the regional Red List assessment [33]).

### Statistical analysis

The effects of crop diversity (CropDiv) and landscape heterogeneity (LandHet) on bird richness were analyzed by applying linear models (total richness) and linear mixed effects models (richness of functional groups; R package ‘nlme’; [40]) R Statistical Software v.3.2.2 [41]. Separate models were fitted for each of the five spatial scales. The scale with the strongest landscape effect was then determined by comparing AICc values of full models. For total richness, fixed factors for each scale-specific model were CropDiv, LandHet and their interaction. To identify guild-specific differences in response, the models assessing effects on species richness of the functional groups (‘Func’) feeding guild, conservation status, habitat preference and nesting behaviour also included the interactions Func x CropDiv and Func x LandHet. Sample size for functional group models varied depending on the number of functional guilds per group (e.g. four feeding guilds in all but one landscapes; Table 2). In these models, ‘study site’ was entered as random term, and variance structures (varIdent) were added for the functional groups feeding guild, conservation status and nesting behavior, to account for variance heterogeneity. All models were fitted using Gaussian distribution as graphical validation of normality and homogeneity of residuals suggested that assumptions for linear models were met. In addition, the complexity of our models and the need to include variance structures justifies the use of Gaussian over Poisson distribution despite the count nature of the data [42]. We did not observe significant spatial autocorrelation of residuals (Moran’s *I* test in R package ‘ape’, all *p*-values > 0.096 [43]). Both landscape variables were *z*-standardized (R package ‘base’, version 3.2.2) to reduce multicollinearity and enhance interpretability of main effects. Model simplification was performed using likelihood ratio-based manual stepwise deletion of non-significant interaction terms. We assessed the significance of fixed effects using F-tests for linear models (total species richness) and Wald chi-square tests for linear mixed effects models with random terms (species richness of functional groups).

**Table 2:** Effects of crop diversity and landscape heterogeneity on bird richness.

	Predictor	Community richness (*n* = 14)				Feeding guild (*n* = 55)			Conservation status (*n* = 41)			Habitat preference (*n* = 28)			Farmland nesters (*n* = 28)		
Scale		*nDF*	*dDF*	*F*	*p*	*DF*	χ^2^	*p*	*DF*	χ^2^	*p*	*DF*	χ^2^	*p*	*DF*	χ^2^	*p*
250m		*R*^*2*^ = 0.3				*R*^*2*^ = 0.98			*R*^*2*^ = 0.92			*R*^*2*^ = 0.35			*R*^*2*^ = 0.91		
	Func	*NA*	*NA*	*NA*	*NA*	3	223.8	**<0.001**	2	335.2	**<0.001**	1	10.08	**0.002**	1	27.12	**<0.001**
	CropDiv	1	11	1.53	0.243	1	0.39	0.535	1	1.43	0.232	1	1.08	0.298	1	1.37	0.242
	LandHet	1	11	5.78	**0.035**	1	1.15	0.284	1	0.14	0.706	1	4.11	**0.043**	1	5.76	**0.016**
	Func x LandHet	*NA*	*NA*	*NA*	*NA*	3	14.25	**0.003**	2	8.84	**0.012**	-	-	**-**	1	3.54	0.06
500m		*R*^*2*^ = 0.45				*R*^*2*^ = 0.98			*R*^*2*^ = 0.91			*R*^*2*^ = 0.45			*R*^*2*^ = 0.89		
	Func	*NA*	*NA*	*NA*	*NA*	3	482.4	**<0.001**	2	450.3	**<0.001**	1	15.83	**0.001**	1	27.12	**<0.001**
	CropDiv	1	11	1.06	0.325	1	0.03	0.859	1	1.05	0.305	1	0.88	0.349	1	1.06	0.304
	LandHet	1	11	6	**0.032**	1	3.64	0.056	1	0.01	0.932	1	0.01	0.987	1	7.89	**0.005**
	Func x LandHet	*NA*	*NA*	*NA*	*NA*	3	27.6	**<0.001**	2	5.73	0.057	1	5.31	**0.021**	1	3.04	0.081
1000m		*R*^*2*^ = 0.42				*R*^*2*^ = 0.97			*R*^*2*^ = 0.91			*R*^*2*^ = 0.5			*R*^*2*^ = 0.9		
	Func	*NA*	*NA*	*NA*	*NA*	3	367.2	**<0.001**	2	301.3	**<0.001**	1	12.98	**<0.001**	1	24.15	**<0.001**
	CropDiv	1	11	1.85	0.201	1	0.78	0.377	1	1.62	0.204	1	1.27	0.261	1	2.56	0.11
	LandHet	1	11	7.73	**0.018**	1	1.67	0.197	1	0.01	0.945	1	0.01	0.983	1	3.94	**0.047**
	Func x LandHet	*NA*	*NA*	*NA*	*NA*	3	32.87	**<0.001**	2	5.27	0.072	1	5.37	**0.02**	-	-	-
2000m		*R*^*2*^ = 0.43				*R*^*2*^ = 0.98			*R*^*2*^ = 0.96			*R*^*2*^ = 0.47			*R*^*2*^ = 0.89		
	Func	*NA*	*NA*	*NA*	*NA*	3	190.7	**<0.001**	2	259.4	**<0.001**	1	12.45	**<0.001**	1	24.15	**<0.001**
	CropDiv	1	11	1.81	0.206	1	0.52	0.47	1	0.05	0.827	1	1.18	0.276	1	1.53	0.216
	LandHet	1	11	10.17	**0.009**	1	2.07	0.15	1	1.93	0.165	1	0.14	0.706	1	2.36	0.125
	Func x LandHet	*NA*	*NA*	*NA*	*NA*	3	37.63	**<0.001**	-	-	**-**	1	4.21	**0.04**	-	-	-
3000m		*R*^*2*^ = 0.34				*R*^*2*^ = 0.97			*R*^*2*^ = 0.97			*R*^*2*^ = 0.41			*R*^*2*^ = 0.85		
	Func	*NA*	*NA*	*NA*	*NA*	3	88.74	**<0.001**	2	248.3	**<0.001**	1	12.31	**<0.001**	1	24.15	**<0.001**
	CropDiv	1	11	0.15	0.707	1	0.01	0.968	1	1.23	0.267	1	0.11	0.739	1	0.03	0.874
	LandHet	1	11	8.55	**0.014**	1	1.44	0.23	1	1.68	0.195	1	6.41	**0.011**	1	0.77	0.381
	Func x LandHet	*NA*	*NA*	*NA*	*NA*	3	22.56	**<0.001**	-	-	**-**	-	-	**-**	-	-	-

Linear models relating total and functional group bird richness to crop diversity (CropDiv), perennial habitat diversity (LandHet, the proxy for landscape heterogeneity) and functional group (Func, only for functional groups models). Two-way interactions between functional group and landscape variables were included in full models yet removed during backwards stepwise model selection if non-significant. The random effect ‘Study site’ was added in functional group models to account for non-independence of samples within study sites. Analysis of nesting behaviour was limited to the farmland bird subset (25 out of 63 species). Significant *p*-values <0.05 are indicated in bold. Model fit was determined with adjusted *R*^*2*^ (total richness) and marginal *R*^*2*^ (richness of functional groups) using the function ‘r.squaredGLMM’ (‘MuMIn’ package). Sample sizes (*n*) varied depending on functional group considered. Significance of fixed effects assessed using F-tests (linear models for total species richness) and Wald chi-square tests (linear mixed effects models with random terms for species richness of functional groups).

In the presence of marginal or significant interactions, we used post hoc multiple comparisons of slopes with manually defined contrast matrices (R package ‘multcomp’, [44]) to determine whether species richness responses of individual functional guilds differed from zero. For this purpose, *p*-values were adjusted for the False Discovery Rate [45]. We repeated the functional groups analyses by excluding guilds with an average of less than three species per field. As the results were qualitatively the same, we thereby confirmed that findings were not affected by the imbalance between highly abundant and rare groups. In addition, this approach highlighted the importance of further investigating individual guild responses in the presence of marginal interactions between functional groups and landscape variables.

Model fit was assessed using adjusted *R*^*2*^ for linear models (total species richness) and marginal *R*^*2*^ (considering fixed effects only) for linear mixed models in functional group analyses (function ‘r.squaredGLMM’ in R ‘MuMIn’ package [46]).

## Results

During four field visits, we observed 63 bird species with a summed total abundance of 1520 individuals. Bird richness varied significantly with the functional group considered (Tables 1, 2 and S1). Insect-feeding and non-farmland bird species were most common, while 17 out of 25 species of farmland birds were non-crop nesters. Although non-threatened birds were most prominent, species listed as endangered and vulnerable on the Bavarian Red List 2016 were recorded in all landscapes, with an average of six species per site encountered during the four visits. The endangered skylark *Alauda arvensis* was the most abundant species (17.8% of observations) and occurred at all sites. The red-listed Eurasian wryneck *Jynx torquilla* and the grey partridge *Perdix perdix* were recorded only once, thereby each accounting for only c. 0.07% of all observations (S2 Table).

### Landscape and scale effects on bird communities

**Fig 1:**
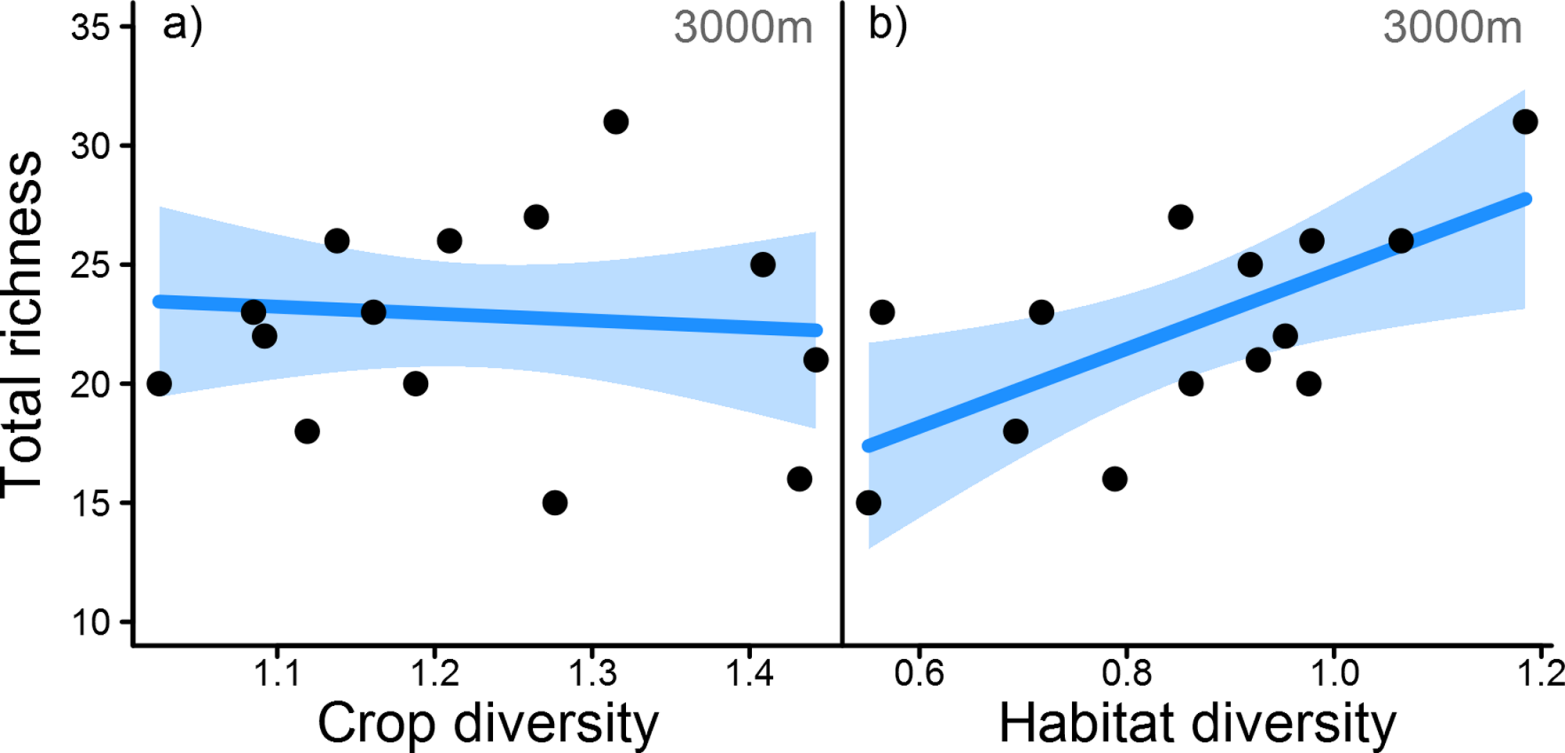
Landscape effects on total bird richness. Effects of a) landscape-level crop diversity (CropDiv) and b) perennial habitat diversity (LandHet, proxy for overall landscape heterogeneity) on total species richness. Exemplified for landscape effects at the 3000 m scale (lowest AICc value) with predicted values for each study site (*n* = 14). Regression line and 95% confidence intervals shown.

Crop diversity did not affect bird communities regardless of the scale or functional group considered (Fig 1 and Table 2). In contrast, landscape heterogeneity enhanced several aspects of bird richness considered in this study. Interactions between crop diversity and landscape heterogeneity were not observed.

**Fig 2:**
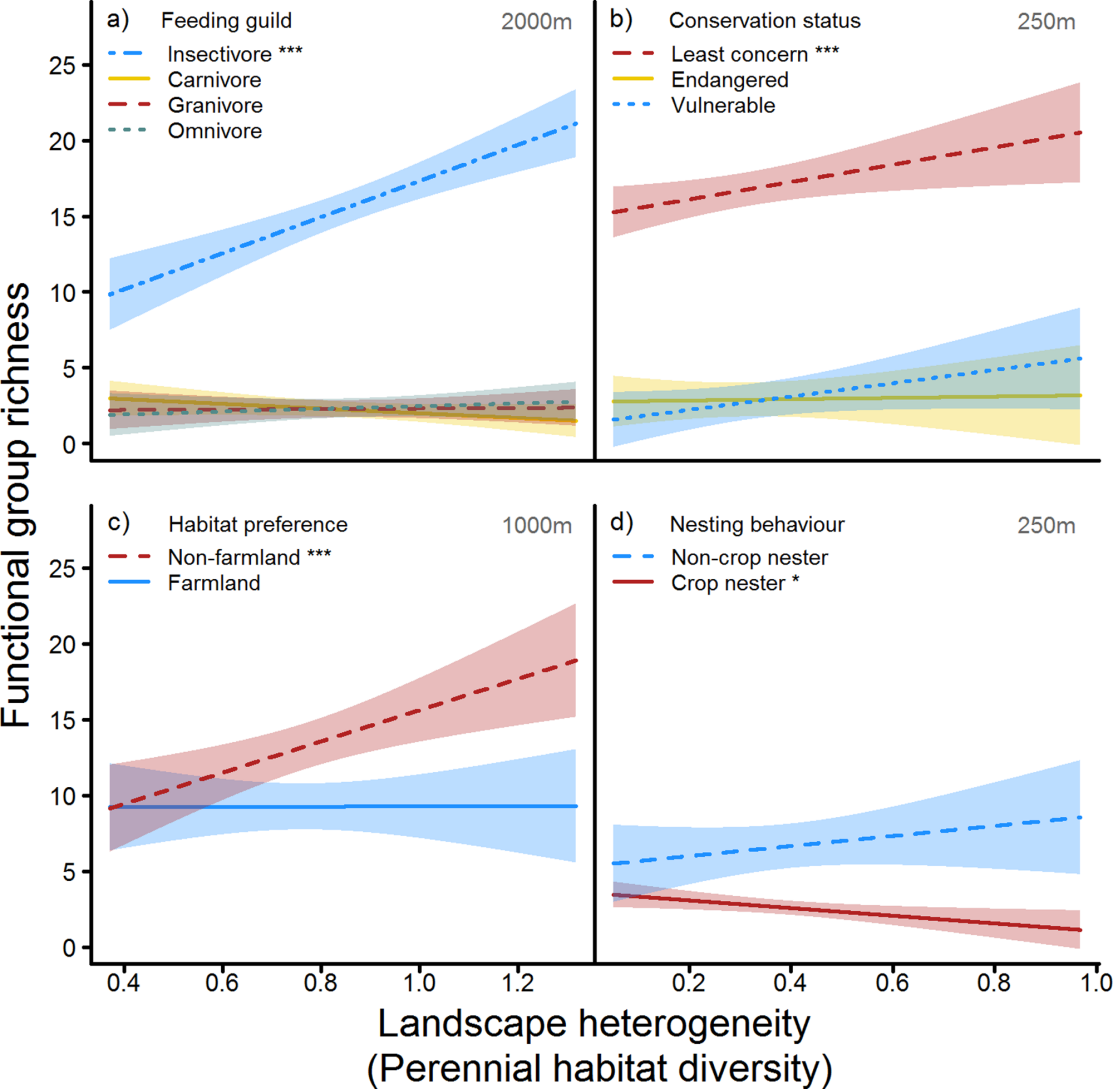
Habitat diversity effects on functional group richness. Effects of perennial habitat diversity (LandHet, proxy for overall landscape heterogeneity) on species richness of the functional groups a) feeding guild (2000m scale), b) conservation status (250m scale), c) habitat preference (1000m scale), and d) nesting behaviour (farmland bird subset, 250m) shown for scales with lowest AICc values. Slopes were tested against zero using contrast matrices with *p*-values adjusted for the False Discovery Rate ([45], S3 Table). Shown are fitted lines and 95% confidence intervals. Signifance levels: *** *p*<0.001, * *p*<0.05.

Extensive landscapes offering a variety of non-crop and perennial habitats, smaller field sizes and lower cropland cover generally harboured the most diverse bird assemblages across all scales (Fig 1A and Table 2). This positive relationship between landscape heterogeneity and total species richness was driven by the response of dominant functional groups such as insectivores, non-farmland birds or species of least conservation concern (Fig 2, Tables 2 and S3). Accordingly, birds preferentially feeding on arthropods were enhanced in extensive landscapes across multiple scales, although the remaining feeding guilds were unaffected (Fig 2A, Tables 2 and S3). Non-threatened birds (‘least concern’ on the regional Red List) were facilitated by landscape heterogeneity at the 250 to 1000 m scale (Fig 2B, Tables 2 and S3). Although functional group x LandHet interactions were only marginal on the larger scales, post hoc comparisons showed strong increases in the species richness of this dominant group, which was confirmed by single-guild analyses. Neither vulnerable nor endangered species showed similar responses. We also observed a positive influence of intermediate-scale landscape heterogeneity on non-farmland birds (500–2000 m scale, Fig 2C, Table 2 and S3). In contrast, the group of farmland specialists showed no benefits of landscape heterogeneity as a whole. However, the differentiation between nesting preference of farmland birds revealed strong reductions of crop-nesting birds at small scales (250-500m), whereas the positive relationship between non-crop nesters and landscape heterogeneity was non-significant due to high inter-field variability (Fig 2D, Tables 2 and S3).

## Discussion

Our study assesses for the first time the individual and interactive effects of crop diversification and landscape heterogeneity on bird species richness and community structure across various spatial scales. We do this by disentangling crop diversity effects from the confounding influence of landscape heterogeneity variables such as perennial habitat diversity, mean field size, seminatural habitat and cropland cover.

Contrary to our hypothesis, we did not observe higher bird species richness in landscapes with diverse cropping systems, regardless of landscape context (low vs. high landscape heterogeneity), functional group or spatial scale considered. Therefore, we cannot confirm previous findings that birds in general or functional groups such as non-crop breeding farmland species in particular benefit from crop functional diversity [15,16,18,22,26,27,39].

The spatial scale of a landscape often determines the outcome of landscape-biodiversity studies [9,19–21]. We overcome this limitation by including a range of local to landscape scales. We also accounted for different within-taxon responses that could mask total richness effects by distinguishing between different functional groups. For example, many farmland bird specialists show negative responses to diversification practices, as they rely on homogeneous systems with large fields and a large share of cereal crops, while non-farmland birds may benefit from the increase of non-crop resources [19,24,25,47]. In our study, however, farmland birds did not decline with crop diversification. These results are in line with studies that found no or very weak effects of crop diversity on farmland birds, when crop diversity measures were separated (uncorrelated) from other aspects of landscape heterogeneity.

The absence of crop diversity-biodiversity relationships in previous studies [14,17,47] suggests that birds may not rely on higher resource amount and continuity presumably provided by crop diversification. This could be the case, if birds do not require crop resources, or are otherwise able to compensate for reduced crop diversity by switching to non-crop resources. Crop diversity may therefore rise in importance in simplified landscapes, were non-crop resources are inadequate [10,18,29]. Despite being located in an intensively farmed area, non-crop habitat cover in our study region was relatively high, and fields small (average amount of seminatural habitat 18.6 ±1.5%, mean patch size 1.6±0.1 ha across study sites and all spatial scales). Accordingly, the mobile bird taxon may not have been as reliant on additional crop resources as in more simplified agroecosystems.

On the other hand, crop-specific pesticide and fertilizer applications, mowing, harvesting or grazing disturbance and other forms of agricultural management could obscure or counteract the benefits of increased resource availability [48]. For instance, a Swedish study reported increased ground beetle diversity with enhanced spatial crop diversity only after accounting for land-use management influences associated with tillage [7]. Negative impacts of chemical intensification on bird diversity have also been reported on the farm scale, especially for ground-breeding farmland birds such as the skylark [21]. Specialist farmland birds are still the most endangered group of birds [13], and although some species respond positively to landscape and non-crop features, local reduction of agricultural intensification may be especially relevant for the conservation of crop-nesting birds [49]. In our case, crop diversity showed a weak, positive trend with the frequency of insecticide application on the study field (Pearson’s *r =* 0.42, p-value = 0.139). Higher rates of local insecticide application in landscapes with greater crop diversity could reduce invertebrate prey of insectivores, the most abundant dietary guild. If local application rates are comparable to farm-scale or regional values, this could explain the slight decline of overall bird richness with diversification on all spatial scales (Fig 1). As we do not have data on landscape-scale insecticide applications, this hypothesis warrants further investigation. However, apart from insecticide-driven reductions, crop-based invertebrate prey in diverse cropping systems may also be reduced due to enhanced insect-mediated pest control [6,50,51], although positive effects of landscape heterogeneity on predators do not always translate to lower prey availability[10]. This may also affect the resource base and thereby the population size and richness of insect-feeding birds.

Lastly, specific crop types may be more important for avian communities, particularly farmland birds, than crop diversity per se. For example, cereals, pastures, set-asides and spring-sown crops have all been linked to changes in total and functional bird species richness, especially for farmland birds [17,18,21,47,52,53]. At the same time, the absolute observed difference in the number of crop types between low and high diversity landscapes was relatively small (difference of four crop types on average across all scales, S1 Table), although focal fields were selected to maximize the range of crop diversity. If additional crops were only grown in low proportions, or increases in crop diversity were driven by a more equal share of a selected number of main crops, then the benefits of crop diversification could be negligible [14].

Either of these explanations of our non-significant findings are possible, yet other reasons are also worthwhile exploring. A taxon like birds, which covers a variety of functionally different and highly mobile species, may require larger spatial scales to detect benefits of crop diversity. For instance, prevalence of significant findings at the largest scale studied may indicate that more significant effects occurred outside the measured range [20]. Alternatively, weak effects of crop diversity (if present) may best be observed using a larger crop diversity gradient, and–due to high between-field variability- a larger sample size.

Opposed to crop diversity, the effects of landscape heterogeneity on bird communities were mainly positive. Our study used perennial habitat diversity as proxy for the overall level of landscape heterogeneity. Due to correlated landscape heterogeneity variables, we emphasize that it is impossible to disentangle the actual driver of the observed positive effects on bird diversity. They could either relate to 1) additional non-crop resources and habitats (resource complementation or niche differentiation [4,54]; 2) increased amounts of seminatural habitat such as field edges for foraging and nesting [55]; 3) smaller field sizes allowing for better access to adjacent non-crop habitats with abundant invertebrate prey [14,15,18,21]; or 4) lower proportions of cropland, another indicator for heterogeneity and potentially reduced overall pesticide application [14,18,21]. Drivers may vary depending on the functional group and scale considered, with scales of response (mainly 250 to 1000 m) comparing well with a previous study identifying the farm as the most relevant management scale for bird conservation purposes [21].

Non-farmland birds include species that rely on forests, settlements or water bodies for nesting and foraging. They are apt to benefit from agricultural extensification and improved resource or habitat availability [25,53], as supported by our results at intermediate scales. The lack of enhancement at the 250 m scale may be due to the study design, which comprised conventionally managed focal fields with simple grass borders and low structural diversity at small spatial scales. Yet even these simple field boundaries and habitats may provide important foraging grounds with abundant prey resources for insectivores, particularly specialist farmland birds such as the skylark [53,55]. Therefore, landscape heterogeneity may favour the diversity of this functional guild independent of the scale considered.

The increase in species richness of the group with the conservation status ‘least concern’ (250 to 1000 m scale) was likely driven by the positive response of insectivores and non-farmland birds, which made up almost 60% and 80% of ‘least concern’ species, respectively. However, the increase was less pronounced than in those guilds, possibly due to some common farmland species, that may have been negatively influenced by high landscape heterogeneity at the cost of cropland habitat and resources. Of the farmland birds, crop-nesters were the only functional guild with declining species richness in heterogeneous landscapes. However, this finding corroborates previous research highlighting the importance of homogeneous, open cropland for some crop-breeding farmland specialists [17], and the potentially detrimental role of field management intensity on this functional group [49].

The remaining functional groups did not show any specific responses to landscape heterogeneity. These groups, including non-insectivores, vulnerable or endangered species and non-crop nesters, may have very specific habitat or resource requirements not met with general diversification efforts [33], and were rarely sampled in our study. For example, the Eurasian wryneck *Jynx torquilla* is more likely to benefit from targeted enhancement of high-value calcareous grasslands than from the extension of other seminatural habitat types [33].

## Conclusion

Three measures build the backbone of Greening, Pillar I of the European Common Agricultural Policy for the period 2015–2020 (CAP, EU Regulation No. 1307/2013), namely 1) retention of permanent grasslands, 2) ecological focus areas, and 3) crop diversification. All are intended to promote sustainable agriculture, biodiversity and ecosystem services, yet only the advantages of grasslands and non-crop habitats have been thoroughly studied. In support of Greening measure one and two, our study confirms that avian diversity, particularly non-farmland species and insectivores, can be enhanced by landscape heterogeneity [2,4,17]. We did not find, however, any benefits of landscape-level crop diversity for bird richness in intensively managed winter wheat systems, in contrast to studies on other taxa (e.g. Carabidae, [7]). Nevertheless, benefits may not only depend on scale, landscape context and functional groups, but also management intensity gradients or interspecific interactions with other agricultural species. This research avenue warrants further investigation. In general, we show that heterogeneity of agricultural landscapes and diversification of non-crop habitats directly benefit overall bird diversity, in addition to targeted, potentially field-based conservation measures aimed at increasing specific nesting and food resources of endangered specialist species.

## Supporting information

S1 Table Description of crop diversity and perennial habitat diversity. Summary statistics of crop diversity (CropDiv) and perennial habitat diversity (LandHet, the proxy for landscape heterogeneity) for different spatial scales. Shown are also the correlation coefficients (Pearson’s *r*) of CropDiv and LandHet with the proportion of cropland (*r* crop), seminatural habitat cover (*r* SNH, including margins along linear elements such as roads and rivers) and average field size *(r* field). For CropDiv, the average number of crop types (and range) at each spatial scale are listed.
(PDF)

S2 Table Bird species classification. List of observed bird species with common name, habitat preference, feeding guild, Red List status (Germany and Bavaria), and total and relative abundance of species across 14 study sites.
(PDF)

S3 Table Effects of perennial habitat diversity on functional groups. Effects of perennial habitat diversity (LandHet, the proxy for landscape heterogeneity) on species richness of functional groups ‘Feeding guild’, ‘Conservation status’ ‘Habitat preference’ and ‘Nesting behaviour’. Only functional groups and spatial scales of models with significant or marginal interactions between LandHet and functional groups are shown. Slopes were tested against zero using contrast matrices and *p*-values of multiple comparisons were adjusted for the False Discovery Rate (Benjamini and Yekutieli, 2001).
(PDF)

S4 Table Excel data file containing total and functional group data used for statistical analysis. (XLSX)
