# Supplementary material for: Landscape heterogeneity rather than crop diversity mediates bird diversity in agricultural landscapes
Source: PLoS One. 2018 Aug 1;13(8):e0200438. doi: 10.1371/journal.pone.0200438 (PMC6070203; doi:10.1371/journal.pone.0200438)
Supplement: S1 Table — Summary statistics of crop diversity (CropDiv) and perennial habitat diversity (LandHet, the proxy for landscape heterogeneity) for different spatial scales. Shown are also the correlation coefficients (Pearson’s r) of CropDiv and LandHet with the proportion of cropland (r crop), seminatural habitat cover (r SNH, including margins along linear elements such as roads and rivers) and average field size (r field). For CropDiv, the average number of crop types (and range) at each spatial scale are listed. (PDF) [file pone.0200438.s001.pdf]

Supporting Information – S1 Table

Landscape heterogenetiy rather than crop diversity mediates bird diversity in agricultural landscapes

Sarah Redlich, Emily A. Martin, Beate Wende, Ingolf Steffan-Dewenter

S1 Table: **Description of crop diversity and landscape heterogeneity.**

Scale	Min	1st Q	Median	Mean	3rd Q	Max	Average crop types	*r* LandHet	*r* crop	*r* SNH	*r* field
CropDiv											
250	0	0.513	0.67	0.686	0.888	1.239	3.3 (1-5)	0.4	-0.12	0.06	-0.58
500	0.5	0.698	0.914	0.915	1.021	1.442	5 (2-8)	0.22	-0.05	-0.24	-0.54
1000	0.74	0.862	1.037	1.056	1.223	1.443	5.4 (2-8)	-0.2	0.32	-0.41	-0.18
2000	0.984	1.056	1.184	1.2	1.332	1.483	8.8 (6-10)	0.05	0.02	-0.35	-0.04
3000	1.025	1.124	1.199	1.225	1.305	1.442	9.4 (8-11)	0.14	0.16	-0.28	-0.24
All scales	0	0.842	1.055	1.016	1.209	1.483	6.38 (1-11)	-	-	-	-
LandHet											
250	0.052	0.228	0.364	0.396	0.574	0.967	-	-	-0.82	0.53	-0.56
500	0.14	0.417	0.53	0.554	0.752	0.89	-	-	-0.92	0.8	-0.5
1000	0.371	0.614	0.727	0.764	0.89	1.319	-	-	-0.93	0.8	-0.5
2000	0.426	0.739	0.848	0.837	0.957	1.224	-	-	-0.96	0.82	-0.58
3000	0.551	0.735	0.89	0.859	0.97	1.185	-	-	-0.93	0.8	-0.7
All scales	0.052	0.436	0.709	0.682	0.899	1.319	-	-	-	-	-

Summary statistics of crop diversity (CropDiv) and perennial habitat diversity (LandHet, the proxy for landscape heterogenetiy) for different spatial scales. Shown are also the correlation coefficients (Pearson's r) of CropDiv and LandHet with the proportion of cropland (r crop), seminatural habitat cover (r SNH, including margins along linear elements such as roads and rivers) and average field size (r field). For CropDiv, the average number of crop types (and range) at each spatial scale are listed.
