# Supplementary material for: Landscape heterogeneity rather than crop diversity mediates bird diversity in agricultural landscapes
Source: PLoS One. 2018 Aug 1;13(8):e0200438. doi: 10.1371/journal.pone.0200438 (PMC6070203; doi:10.1371/journal.pone.0200438)
Supplement: S2 Table — List of observed bird species with common name, habitat preference, feeding guild, Red List status (Germany and Bavaria), and total and relative abundance of species across 14 study sites. (PDF) [file pone.0200438.s002.pdf]

Supporting Information - S2 Table

Landscape heterogeneity rather than crop diversity mediates bird diversity in agricultural landscapes

Sarah Redlich, Emily A. Martin, Beate Wende, Ingolf Steffan-Dewenter

S2 Table: Bird species classification.

Species	Common name	Feeding guild[1]	Conservation status[2]	Habitat preference[3]	Nesting behaviour[4]	Total abundance	Relative abundance
Acrocephalus scirpaceus	Eurasian reed-warbler	Insectivore	Least concern	Non-Farmland	Non-crop	5	0.33
Alauda arvensis	Eurasian skylark	Insectivore	Endangered	Farmland	**Crop**	271	17.83
Anas platyrhynchos	Mallard duck	Omnivore	Least concern	Non-Farmland	Non-crop	9	0.59
Apus apus	Common swift	Insectivore	Endangered	Farmland	**Non-crop**	9	0.59
Ardea cinerea	Grey heron	Carnivore	Vulnerable	Non-Farmland	Non-crop	2	0.13
Aythya fuligula	Tufted duck	Omnivore	Least concern	Non-Farmland	Non-crop	2	0.13
Buteo buteo	Common buzzard	Carnivore	Least concern	Non-Farmland	Non-crop	18	1.18
Carduelis cannabina	Common linnet	Granivore	Endangered	Farmland	**Non-crop**	2	0.13
Carduelis carduelis	European goldfinch	Granivore	Vulnerable	Farmland	**Non-crop**	2	0.13
Carduelis chloris	European greenfinch	Granivore	Least concern	Farmland	**Non-crop**	9	0.59
Certhia brachydactyla	Short-toed treecreeper	Insectivore	Least concern	Non-Farmland	Non-crop	1	0.07
Circus aeruginosus	Marsh harrier	Carnivore	Least concern	Farmland	**Crop**	1	0.07
Circus cyaneus	Hen harrier	Carnivore	Endangered	Non-Farmland	Non-crop	4	0.26
Circus pygargus	Montagu's harrier	Carnivore	Endangered	Farmland	**Crop**	5	0.33
Columba livia	Rock pigeon	Granivore	Least concern	Farmland	**Non-crop**	12	0.79
Columba palumbus	Common wood pigeon	Granivore	Least concern	Non-Farmland	Non-crop	92	6.05
Corvus corone	Carrion Crow	Omnivore	Least concern	Non-Farmland	Non-crop	2	0.13
Corvus frugilegus	Rook	Omnivore	Least concern	Farmland	**Non-crop**	45	2.96
Coturnix coturnix	Common quail	Granivore	Endangered	Farmland	**Crop**	3	0.20
Cuculus canorus	Common Cuckoo	Insectivore	Vulnerable	Non-Farmland	Non-crop	14	0.92
Delichon urbicum	Common house martin	Insectivore	Endangered	Farmland	**Non-crop**	70	4.61

Dendrocopos major	Great spotted woodpecker	Insectivore	Least concern	Non-Farmland	Non-crop	2	0.13
Emberiza calandra	Corn bunting	Granivore	Endangered	Farmland	**Crop**	4	0.26
Emberiza citrinella	Yellowhammer	Insectivore	Least concern	Farmland	**Non-crop**	125	8.22
Emberiza schoeniclus	Common reed bunting	Granivore	Least concern	Non-Farmland	Non-crop	1	0.07
Erithacus rubecula	European robin	Insectivore	Least concern	Non-Farmland	Non-crop	7	0.46
Falco tinnunculus	Common kestrel	Carnivore	Least concern	Farmland	**Non-crop**	16	1.05
Fringilla coelebs	Common chaffinch	Insectivore	Least concern	Non-Farmland	Non-crop	34	2.24
Garrulus glandarius	Eurasian jay	Omnivore	Least concern	Non-Farmland	Non-crop	5	0.33
Hirundo rustica	Barn swallow	Insectivore	Vulnerable	Farmland	**Non-crop**	27	1.78
Jynx torquilla	Eurasian wryneck	Insectivore	Endangered	Non-Farmland	Non-crop	1	0.07
Lanius collurio	Red-backed shrike	Insectivore	Vulnerable	Farmland	**Non-crop**	8	0.53
Luscinia megarhynchos	Common nightingale	Insectivore	Least concern	Non-Farmland	Non-crop	9	0.59
Luscinia svecica	Bluethroat	Insectivore	Least concern	Non-Farmland	Non-crop	1	0.07
Milvus milvus	Red kite	Carnivore	Vulnerable	Non-Farmland	Non-crop	3	0.20
Motacilla alba	White wagtail	Insectivore	Least concern	Farmland	**Non-crop**	44	2.89
Motacilla flava	Yellow wagtail	Insectivore	Least concern	Farmland	**Crop**	81	5.33
Oriolus oriolus	Eurasian golden oriole	Insectivore	Vulnerable	Non-Farmland	Non-crop	10	0.66
Parus ater	Coal tit	Omnivore	Least concern	Non-Farmland	Non-crop	3	0.20
Parus caeruleus	Eurasian Blue Tit	Insectivore	Least concern	Non-Farmland	Non-crop	74	4.87
Parus major	Great tit	Insectivore	Least concern	Non-Farmland	Non-crop	105	6.91
Parus palustris	Marsh tit	Insectivore	Least concern	Non-Farmland	Non-crop	6	0.39
Passer domesticus	House sparrow	Granivore	Vulnerable	Farmland	**Non-crop**	1	0.07
Passer montanus	Eurasian tree sparrow	Insectivore	Vulnerable	Farmland	**Non-crop**	9	0.59
Perdix perdix	Grey partridge	Granivore	Endangered	Farmland	**Crop**	1	0.07
Phasianus colchicus	Common pheasant	Omnivore	Least concern	Farmland	**Crop**	9	0.59
Phoenicurus ochruros	Black redstart	Insectivore	Least concern	Non-Farmland	Non-crop	8	0.53
Phoenicurus phoenicurus	Common redstart	Insectivore	Endangered	Non-Farmland	Non-crop	9	0.59
Phylloscopus collybita	Common chiffchaff	Insectivore	Least concern	Non-Farmland	Non-crop	24	1.58
Phylloscopus trochilus	Willow warbler	Insectivore	Least concern	Non-Farmland	Non-crop	8	0.53
Pica pica	Common magpie	Omnivore	Least concern	Non-Farmland	Non-crop	26	1.71
Picus viridis	European green woodpecker	Insectivore	Least concern	Non-Farmland	Non-crop	9	0.59
Prunella modularis	Dunnock	Insectivore	Least concern	Non-Farmland	Non-crop	1	0.07
Serinus serinus	European serin	Granivore	Least concern	Farmland	**Non-crop**	4	0.26
Sitta europaea	Eurasian nuthatch	Insectivore	Least concern	Non-Farmland	Non-crop	6	0.39
Streptopelia decaocto	Eurasian collared dove	Granivore	Least concern	Non-Farmland	Non-crop	6	0.39
Sturnus vulgaris	Common starling	Insectivore	Least concern	Farmland	**Non-crop**	96	6.32
Sylvia atricapilla	Blackcap	Insectivore	Least concern	Non-Farmland	Non-crop	20	1.32
Sylvia borin	Garden warbler	Insectivore	Least concern	Non-Farmland	Non-crop	6	0.39
Sylvia communis	Common whitethroat	Insectivore	Vulnerable	Farmland	**Non-crop**	25	1.64
Turdus merula	Common blackbird	Insectivore	Least concern	Non-Farmland	Non-crop	89	5.86

Turdus philomelos	Song thrush	Insectivore	Least concern	Non-Farmland	Non-crop	14	0.92
Turdus pilaris	Fieldfare	Omnivore	Least concern	Non-Farmland	Non-crop	5	0.33
Total						*1520*	*100*

List of observed bird species with common name, habitat preference, feeding guild, Red List status (Germany and Bavaria), and total and relative abundance of species across 14 study sites.

[1] Feeding guild classification based on (Chiron et al., 2014) and (Henderson et al., 2009)

[2] Classification according to the Bavarian Red List for birds (Bayerisches Landesamt für Umwelt, 2016): least concern, vulnerable, endangered (including very rare, near endangered, highly endangered and regionally extinct species).

[3] Classification based on EBBC (EBBC, 2015) with farmland birds defined as species of which >50% of the population use agricultural land for breeding or feeding

[4] Classification of farmland bird species based on (Hiron et al., 2015; Josefsson et al., 2016). A subset only including farmland birds (in bold) was used to test for differences in bird richness related to nesting habits of birds primarily utilizing cropland (farmland birds).

References

Bayerisches Landesamt für Umwelt. Rote Liste und Liste der Brutvögel Bayerns. 2016.

EBBC. European Bird Census Council [Internet]. 2015. Available from: http://www.ebcc.info

Henderson IG, Ravenscroft N, Smith G, Holloway S. Effects of crop diversification and low pesticide inputs on bird populations on arable land. Agric Ecosyst Environ. 2009 Jan;129(1–3):149–56.

Hiron M, Berg Å, Eggers S, Berggren Å, Josefsson J, Pärt T. The relationship of bird diversity to crop and non-crop heterogeneity in agricultural landscapes. Landsc Ecol. 2015 Dec 1;30(10):2001–13.

Josefsson J, Berg Å, Hiron M, Pärt T, Eggers S. Sensitivity of the farmland bird community to crop diversification in Sweden: does the CAP fit? J Appl Ecol. 2016 Sep 1; 518-526.
