# Supplementary material for: Landscape heterogeneity rather than crop diversity mediates bird diversity in agricultural landscapes
Source: PLoS One. 2018 Aug 1;13(8):e0200438. doi: 10.1371/journal.pone.0200438 (PMC6070203; doi:10.1371/journal.pone.0200438)
Supplement: S3 Table — Effects of perennial habitat diversity (LandHet, the proxy for landscape heterogeneity) on species richness of functional groups ‘Feeding guild’, ‘Conservation status’ ‘Habitat preference’ and ‘Nesting behaviour’. Only functional groups and spatial scales of models with significant or marginal interactions between LandHet and functional groups are shown. Slopes were tested against zero using contrast matrices and p-values of multiple comparisons were adjusted for the False Discovery Rate (Benjamini and Yekutieli, 2001). (PDF) [file pone.0200438.s003.pdf]

Supporting Information – S3 Table

Landscape heterogeneity rather than crop diversity mediates bird diversity in agricultural landscapes

Sarah Redlich, Emily A. Martin, Beate Wende, Ingolf Steffan-Dewenter

S3 Table: Effects of landscape heterogeneity on functional groups.

Model	Estimate	SE	z-value	*p*-value	
Feeding guild					
250m					
LandHet Carnivore vs Zero	-0.34	0.32	-1.07	0.348	
LandHet Granivore vs Zero	0.18	0.35	0.53	0.593	
LandHet Insectivore vs Zero	2.54	0.97	2.63	**0.029**	*
LandHet Omnivore vs Zero	0.55	0.36	1.54	0.177	
500m					
LandHet Carnivore vs Zero	-0.63	0.33	-1.91	0.094	
LandHet Granivore vs Zero	0.02	0.4	0.05	0.957	
LandHet Insectivore vs Zero	3.4	0.85	4.01	**<0.001**	***
LandHet Omnivore vs Zero	0.29	0.41	0.7	0.601	
1000m					
LandHet Carnivore vs Zero	-0.44	0.34	-1.29	0.328	
LandHet Granivore vs Zero	-0.12	0.35	-0.34	0.737	
LandHet Insectivore vs Zero	3.47	0.69	5.05	**<0.001**	***
LandHet Omnivore vs Zero	0.3	0.39	0.78	0.485	
2000m					
LandHet Carnivore vs Zero	-0.57	0.39	-1.44	0.214	
LandHet Granivore vs Zero	0.06	0.43	0.14	0.889	
LandHet Insectivore vs Zero	4.36	0.81	5.39	**<0.001**	***
LandHet Omnivore vs Zero	0.33	0.47	0.7	0.602	
3000m					
LandHet Carnivore vs Zero	-0.56	0.47	-1.2	0.328	
LandHet Granivore vs Zero	0.33	0.5	0.66	0.634	
LandHet Insectivore vs Zero	4.67	1.13	4.12	**<0.001**	***
LandHet Omnivore vs Zero	0.31	0.57	0.55	0.648	
Conservation status					
250m					
LandHet Endangered vs zero	-0.26	0.7	-0.38	0.706	
LandHetI Least concern vs zero	2.4	0.7	3.42	**0.004**	**
LandHet Vulnerable vs zero	1.13	0.76	1.49	0.206	
500m					
LandHet Endangered vs zero	-0.06	0.73	-0.09	0.932	

LandHetI Least concern vs zero	2.29	0.73	3.13	**0.011**	*
LandHet Vulnerable vs zero	0.94	0.84	1.12	0.392	
1000m					
LandHet Endangered vs zero	0.05	0.66	0.07	0.945	
LandHetI Least concern vs zero	2.16	0.66	3.26	**0.007**	**
LandHet Vulnerable vs zero	0.81	0.74	1.1	0.408	
Habitat preference					
500m					
LandHet Farmland vs zero	-0.02	1.07	-0.02	0.987	
LandHet Non-farmland vs zero	3.43	1.07	3.2	**0.004**	**
1000m					
LandHet Farmland vs zero	0.02	0.97	0.02	0.983	
LandHet Non-farmland vs zero	3.17	0.97	3.27	**0.003**	**
2000m					
LandHet Farmland vs zero	0.45	1.2	0.38	0.706	
LandHet Non-farmland vs zero	3.92	1.2	3.28	**0.003**	**
Nesting behaviour					
250m					
LandHet Crop vs zero	-0.78	0.32	-2.4	**0.033**	*
LandHet Non-crop vs zero	1.02	0.95	1.08	0.282	
500m					
LandHet Crop vs zero	-0.9	0.32	-2.81	**0.01**	*
LandHet Non-crop vs zero	0.87	1	0.87	0.384	

Effects of perennial habitat diversity (LandHet, the proxy for non-crop landscape extensification) on species richness of functional groups ‘Feeding guild’, ‘Conservation status’ ‘Habitat preference’ and ‘Nesting behaviour’. Only functional groups and spatial scales of models with significant or marginal interactions between LandHet and functional groups are shown. Slopes were tested against zero using contrast matrices and *p*-values of multiple comparisons were adjusted for the False Discovery Rate (Benjamini and Yekutieli, 2001). Significant *p*-values are indicated in bold and marked with asterisks: (*) $p<0.1$, * $p<0.05$, ** $p<0.01$, *** $p<0.001$.

References

Benjamini Y, Yekutieli D. The control of the false discovery rate in multiple testing under dependency. Ann Stat. August 2001;29(4):1165–88.Chiron, F., Chargé, R., Julliard, R., Jiguet, F., Muratet, A., 2014. Pesticide doses, landscape structure and their relative effects on farmland birds. Agric. Ecosyst. Environ. 185, 153–160. doi:10.1016/j.agee.2013.12.013
